# Understanding the Unmet Accommodation Needs of People Working with Mental or Cognitive Conditions: The Importance of Gender, Gendered Work, and Employment Factors

**DOI:** 10.1007/s10926-023-10132-4

**Published:** 2023-10-25

**Authors:** Geneviève Jessiman-Perreault, Monique A. M. Gignac, Aaron Thompson, Peter M. Smith

**Affiliations:** 1 Institute for Work and Health, 400 University Ave Suite 1800, Toronto, ON M5G 1S5 Canada; 2 Dalla Lana School of Public Health, University of Toronto, 155 College St Room 500, Toronto, ON M5T 3M7 Canada; 3 Workplace Safety and Insurance Board, 200 Front Street West, Toronto, ON M5V 3J1 Canada; 4 Faculty of Medicine, University of Toronto, 1 King’s College Cir, Toronto, ON M5S 1A8 Canada; 5 School of Public Health and Preventive Medicine, Monash University, Wellington Rd, Clayton, VIC 3800 Australia

**Keywords:** Employment, Mental Health, Accommodations, Disability, Gender

## Abstract

**Purpose:**

Workplace support needs for women and men living with mental health conditions are not well understood. This study examined workplace accommodation and support needs among women and men with and without mental health or cognitive conditions and individual and workplace factors associated with having unmet needs.

**Methods:**

A cross-sectional survey of 3068 Canadian workers collected information on disability, gender, gendered occupations, job conditions, work contexts, and workplace accommodations. Multivariable logistic regression analyses examined gender- and disability-based differences in unmet needs for workplace flexibility, work modifications, and health benefits, and the association of work context (i.e., work schedule, job sector) and job conditions (i.e., precarious work) on the likelihood of unmet accommodation needs. The additive (i.e., super- or sub-additive) and multiplicative effects of disability, gender, and occupational gender distribution on the probability of unmet accommodation needs were also assessed.

**Results:**

The most common unmet workplace accommodation was work modifications reported by 35.9% of respondents with mental/cognitive disability and workplace flexibility reported by 19.6% of individuals without a mental/cognitive disability. Women, employees in female dominant occupations, and participants with mental/cognitive disabilities were more likely to report unmet needs compared with men, employees in non-female dominant occupations, and participants without disabilities but these findings were largely explained by differences in job conditions and work contexts. No interacting effects on the likelihood of reporting unmet needs for workplace accommodations were observed.

**Conclusions:**

To support employee mental health, attention is needed to address work contexts and job conditions, especially for people working with mental/cognitive disabilities, women, and workers in female-dominated occupations where unmet accommodation needs are greatest.

**Supplementary Information:**

The online version contains supplementary material available at 10.1007/s10926-023-10132-4.

## Background

Employee mental health is an important occupational issue [1–5], increasingly recognized in the wake of the COVID-19 pandemic [6–9]. Working-aged Canadians with disabilities experience employment rates that are about 21% lower than Canadians without disabilities [10]. However, evidence indicates that most people living with disabilities want to work and have the capacity to do so [11]. Evidence also suggests that workplace accommodations help people living with mental and/or cognitive conditions retain employment and improve some mental health symptoms [12, 13]. For example, a systematic review conducted by Zafar et al. [12] reported that modifications to the work environment for employees living with mental health disorders improved length of job tenure and reduced severity of mental health symptoms. Similarly, Maesta et al. [13] demonstrated individuals with mental health conditions who received workplace accommodations such as work modifications and workplace flexibility were more likely to remain in the workplace four years later.

In Canada, employers have a legal duty to accommodate employees who fall into 13 protected classes, including disability, unless the employer can show that the accommodation would cause undue hardship [14]. In addition, the Accessible Canada Act, aims to proactively identify and remove barriers that prevent accessibility across seven areas, including the workplace. Under this Act, regulated workplaces are required to publish their accessibility plans, consult with stakeholders for feedback, and report on their progress [15]. Research has found that individuals working with a disability often need a range of workplace accommodations and supports, rather than one single accommodation [16–20]. Yet, in Canada, as well as globally, employers often struggle to provide best practice accommodations to individuals with mental or cognitive disabling conditions [21], due to a number of factors including; a lack of awareness of what constitutes an accommodation for people with mental or cognitive disabilities [22], discrimination from the employer or a poor workplace culture [23], and a perception that accommodations for people with mental or cognitive disabling conditions were less reasonable [24]. Therefore, it is important to examine accommodation needs across different accommodation types among people living with mental or cognitive disabling conditions. Accommodation types include efforts to make working more flexible (e.g., flexible scheduling of start and finish times, work-at-home arrangements), modifications to job duties or the work environment (e.g., workstation changes), and providing health benefits at work (e.g., prescription drug coverage and psychological services).

While there is ample evidence supporting the benefits of work accommodations and supports [12, 13], and legislation supporting employees’ right to request reasonable accommodations, there is emerging evidence that many workers with mental health conditions (e.g., anxiety and depressive disorder) [25] and cognitive conditions (e.g., attention-deficit/hyperactivity disorder and autism) causing disability [26] have high levels of unmet accommodation needs which exceed those without conditions causing disability and those with physical conditions causing disability [20, 27]. While there has been some examination of the association between job conditions, work context and workplace accommodation needs among individuals employed with physical conditions, research to date has not extensively explored the individual, work context (e.g., work hours, job sector), and job condition (e.g., precarious work) factors associated with having unmet accommodation needs among people working with mental/cognitive disabling conditions. Research with workers with physical conditions causing disability at work (e.g., arthritis) has found that workers in the education, health, sciences, arts, sales, and retail job sectors, who worked part-time, and reported greater job stress and lower job control were more likely to be associated with unmet accommodation needs [28]. In a study of individuals with mobility limitations, Balser et al. [29] found that employment in the non-profit sector was associated with being less likely to receive accommodations altering the physical work environment, and that union membership was associated with being less likely to receive assistive technologies and work at home accommodations. People living with disabling conditions also are more likely to be employed in precarious work arrangements [28–31], even after controlling for factors such as education [32], which may also result in individuals being less likely to have workplace accommodation needs met [33] and more likely to have limited benefits [34].

Gender may also be an important factor because women report greater accommodation needs and a greater number of unmet workplace accommodation needs compared with men [28]. This association may be partially due to job conditions and work contexts as women are more likely to be employed in precarious work arrangements [35, 36] and are more likely to experience job insecurity compared with men [37], which is associated with unmet accommodation needs [33] and limited benefits and union protections [34, 35].

Given the higher risks of having unmet accommodation needs among women, as well as among people living with disabilities, it is important to examine how these two factors interact. One previous study found that female sex and disability had an interacting effect on the odds of reporting unmet workplace accommodation needs [28], with women with and without disabilities having higher odds of unmet workplace support needs, compared with men with no disabilities. The intersection between sex, gender and disability requires further examination, especially to consider aspects of the workplace context and job conditions. Studies to date have primarily focused on measuring the effects of sex and gender using a binary variable (women versus men). To gain a better understanding of the role of gender in work and health, it may be important to account for differences in jobs and work tasks, particularly when considering how the segregation of men and women that has traditionally existed in some occupations [38]. To date, Glauber [39] found gender-neutral occupations offered greater opportunities for flexible work compared to male-dominant or female-dominant occupations. However, studies have not explored the potential intersecting effect of being an individual with a disabling condition and employment in occupations with different gendered distributions (i.e., gender-neutral, female-dominant, and male-dominant occupations).

In addition to gendered work, other dimensions of work context and job conditions are important factors influencing whether workplace accommodation needs are met. Past research indicates that workers in precarious work arrangements (i.e., part-time, contract or gig work) are less likely to receive needed accommodations [33]. This relationship between precarious work and unmet accommodation needs has also been demonstrated among people living with disabling conditions [34, 40]. In addition, some research on older workers with a physical disability has found that variables like physically demanding work, job control, and work stress are more important than health factors in understanding unmet accommodation needs [41]. Yet, although many job conditions (e.g., precarious work) and work contexts (e.g., part-time work) that have been found to individually impact women [36, 37, 42] and people living with mental/cognitive conditions causing disability [30, 32, 33], research to date has not examined these factors in the context of availability of workplace accommodations and supports. Therefore, it is important to understand the role of these work contexts and job conditions on observed gender- and disability-based differences in unmet needs for accommodations.

To address these gaps, the objectives of this study are to (1) compare the unmet accommodation needs among women and men with and without mental/cognitive conditions causing disability; (2) explore the effect of gender identity (i.e., self-identifying as being male or female), occupational gender distribution and mental/cognitive disability on unmet workplace accommodation needs after controlling for workplace context and job control factors, and (3) explore whether the combination of two measures of gender (i.e., gender identity and occupational gender distribution) and mental/cognitive disability have an additive or multiplicative impact on unmet workplace accommodation needs.

## Methods

### Study Design and Recruitment

An online, cross-sectional survey was administered to a sample of Canadians in May-June 2020. The questionnaire was available in English and French. Study sampling included individuals working with a chronic physical or mental health condition that caused disability at least some of the time at work. No information was collected distinguishing between participants whose health conditions might have been contributed to or caused by their jobs and those that were not work related as these attributions are difficult to make and often disputed by employers. The questionnaire was administered by a survey research firm that maintains a panel of about 100,000 Canadians assembled to match the demographic characteristics of the Canadian population. Consent to participate was received from all participants. Ethics approval was received from the University of Toronto Research Ethics Board [REB#39085].

### Inclusion and Exclusion Criteria

Inclusion criteria were being 18 years of age or older and currently employed at least 12 h per week or employed for at least 3 months in the previous year. Due to the focus on mental/cognitive conditions, respondents living with only a physical disability (n = 406) were excluded from analyses, as were self-employed respondents (n = 69) who do not receive employer-provided accommodations. Respondents who were not currently working at the time of the survey (i.e., “unemployed but looking for work”, “unemployed and not looking for work”, “retired”, “staying at home to care for my family”, and “student”) were excluded from analyses (n = 286).

### Outcome Measures

#### Workplace Accommodations and Supports

Respondents were asked, “Which of the following benefits, policies or workplace accommodations do you need to use?” Six types of accommodations or supports were provided addressing modifications to the work environment (e.g., modifications to job duties, workstation adaptations), workplace flexibility (e.g., flexible scheduling, work-at-home) and health benefits (e.g., prescription drug coverage, access to comprehensive medical services). If a respondent indicated they needed workplace accommodation, but it was not available to them they were coded as having “unmet needs”. Respondents needing accommodation or support that was available to them, were coded as “needs met”, and those not needing the accommodation were coded as “no needs”.

### Independent Variables

#### Gender Identity and Occupational Gender Distribution

Participants were asked, “How do you self-identify in terms of gender?” Responses were: (1) man, (2) woman, (3) I do not identify within the gender binary, and (4) I prefer not to disclose information concerning my gender. Due to a low sample size (i.e., less than 10), respondents who did not identify within the gender binary or did not disclose their gender were marked as missing on this variable. Occupational gender distribution assessed gender in the context of occupational segregation (i.e., the tendency of men and women to work in different occupations) [43]. This was done by coding occupation into 47 groups using the National Occupational Classification System [44] and then, drawing on methods established by Smith and Koehoorn [45], creating a three-level variable: female-dominant (< 33% male), gender-neutral (33-66% male), and male-dominant (> 66% male) based on occupational segregation reported in the Canadian Labour Force Survey from May to July 2020 [46–48].

#### Conditions Creating Disability

Questions were adapted from the short disability screening questionnaire (DSQ) designed by Statistics Canada to measure disability at work [49]. The DSQ aligns with the International Classification of Functioning, Disability, and Health (ICF) [50]. The DSQ questions were modified to focus on employment. Respondents were asked: 1) “Do you have any physical condition that can make working difficult at least some of the time”, 2) “ Do you have any mental or cognitive condition that can make working difficult at least some of the time?”, and 3) “Do you have any other health problem(s) or a long-term condition(s) that has lasted or is expected to last for six months or more that can make working difficult at least some of the time?”. If the respondent answered “yes” to any of the 3 questions they were asked to specify the condition or health problem. Disability type was coded as (1) no disability, (2) physical disability, (3) mental and/or cognitive disability, and (4) both physical and mental and/or cognitive disability.

#### Covariates

Covariates were grouped into individual factors (i.e., age, education), work context (i.e., union membership, work schedule, job status, industry, workplace size), and job conditions. (i.e., physical job demands, psychological job demands, job control, job insecurity, perceived temporariness, precariousness of wages, vulnerability). Variable creation is briefly described in the following section with further details outlined in Table 1.

**Table 1:** Definition and Coding of Study Covariates

Variable Category	Variable	Survey Question	Coding
Individual Factors	Age	In what year were you born?	Numeric
	Education	What is the highest level of education you have received?	• High school diploma or less • Some college/university • Post-secondary degree/diploma
Work Context	Union membership	Do you belong to a union or a professional/managerial society at your place of employment that acts as a bargaining unit	• Yes • No
	Work schedule	In the past year, which of the following best describes your work schedule?	• Regular daytime schedule • Non-standard work hours
	Job status	Which category best fits your current job status?	• Employed full-time • Employed part-time • On short-term leave or temporarily laid off
	Industry	What job sector or industry do you work in?	• Banking, insurance, business, technology, and professional services • Education, health sciences, art, social sciences, and non-profit • Government • Sales, retail, and hospitality • Construction, transport, utilities, manufacturing, agriculture, mining, and logging
	Workplace size	In total, how many employees work at your organization at the location where you work?	• Small (1–99 employees) • Medium (100–499 employees) • Large (500 or more employees)
Job Conditions	Physical job demands	To what extent does your work involve physical activity or movement (e.g., bending, lifting, walking, etc.)	• Not at all • A little • Somewhat • Quite a bit • A great deal
	Psychological job demands	To what extent is your work mentally demanding (e.g., fast-paced, many deadlines, multiple tasks to juggle, cognitively difficult, emotionally challenging)?	• Not at all • A little • Somewhat • Quite a bit • A great deal
	Job control	In general, how much overall control do you have over your work activities and work-related matters	• Very little • Little • A moderate amount • Much • Very much
	Job insecurity	Derived variables based on responses to 2 questions: 1) Are you worried about becoming unemployed? 2) Are you worried about it being difficult for you to find another job if you become unemployed?	0–4, where 0 is the least perceived job insecurity and 4 is the most perceived job insecurity.
	Perceived temporariness	Derived variables based on responses to 2 questions: 1) Do you have a permanent position with your employer or are you on a contract? 2a) If on contract, how long is your current contract? 2b) How long have you worked for your current employer/ been self-employed?	0–4, where 0 is the least perceived temporariness and 4 is the most perceived temporariness.
	Precariousness of wages	Derived variables based on responses to 3 questions: 1) Please check the box for the range that comes closest to your total household income before taxes, in the past 12 months. 2) Do your current wages or salary allow you to cover your basic expenses? 3) Do your current wages or salary allow you to cover unexpected expenses?	0–4, where 0 is the least precarious wages and 4 is the most precarious wages.
	Vulnerability	Derived variables based on responses to 3 questions: 1) Are you able to ask for better working conditions without concern or retaliation or punishment? 2) Would your current employment be negatively affected if you raised a health and safety concern or raised an employment rights concern with your employer(s)? 3) To what extent do you feel that your organization is supportive of your personal needs?	0–4, where 0 is the least vulnerability and 4 is the most vulnerability.

#### Individual Factors

Information was collected on age (year of birth) and education (“what is the highest level of education you have received?” – grouped into high school diploma or less, some college/university, and post-secondary degree/diploma).

#### Work Context

Respondents were asked whether they belonged to a union or bargaining unit and responses were coded as a binary (yes/no) variable. Respondents were asked about their work schedule with responses coded as a “regular daytime schedule” or “non-standard work hours”. Job status was measured as, (1) “employed full-time”, (2) “employed part-time”, and (3) “on short-term leave or temporarily laid off”. Job sector was measured using 23 categories from the North American Industry Classification System (NAICS) [51], which were collapsed into 5 categories. A question about workplace size divided responses into 3 categories to align with federal classification of workplace size (small, medium, and large) [52].

#### Job Conditions

Seven items provided information on respondents’ subjective perceptions of their work and workplace. The first three items asked respondents about their perceived physical and psychological demands and their perceived job control. Responses were captured on a five-point Likert scale (not at all, a little, somewhat, quite a bit, a great deal). The four remaining items were generated based on results from a factor analysis that included 10 questions and generated 4 items: perceived job insecurity, temporariness of work, precariousness of wages, and vulnerability of work. For each item, scores across component questions were averaged across the number of variables each respondent answered from each item to provide each item with a single score from 0 to 4, where 0 represented the least amount of insecurity, temporariness, precariousness or vulnerability and 4 represented the most.

### Analysis

Factor analysis was conducted with 10 variables focused on job conditions using an oblique rotation to allow for correlations between factors to conceptually organize additional items assessing perceived job conditions. A factor loading of 0.4 was used as a cut-point for the acceptability of an item loading on a factor [53]. Results from the analysis generated four factors: perceived job insecurity, temporariness of work, precariousness of wages, and vulnerability of work (results not shown but available upon request). These four factors were extracted from the factor analysis and compared with our generated variables (as described in the measures section) and found high correlations between the factors extracted and the generated variable, therefore generated variables were used for subsequent analysis to allow for easier interpretation.

Sample characteristics were examined using percentages and frequencies for categorical variables and mean and standard deviations for numeric variables. Similarities and differences in sample characteristics by the presence of a mental/cognitive disabling condition or no disability were examined using chi-square and t-tests. Bivariate analysis was then conducted for three outcome variables: (1) workplace flexibility; (2) work modifications and (3) health benefits by mental/cognitive disabling condition using chi-square tests.

Multivariable logistic regression analyses assessed the relationship between the study variables and the outcome variables: workplace flexibility; work modifications; and health benefits. Additive and multiplicative interactions were examined for gender and mental/cognitive disabling conditions, and occupational gender distribution and mental/cognitive disabling conditions. Additive interactions assessed the extent to which combinations of exposures were synergistic, or sub-additive (i.e., greater than if adding the risks associated with each exposure independently), while multiplicative interactions assessed the extent to which exposure to outcome relationships differed across the levels of a third measure.

Additive interactions were assessed using the Synergy Index (SI), using the approach outlined by Andersson et al. [54], which has been shown to be optimal in adjusted analyses compared to the attributable proportion due to interaction or the relative excess risk due to interaction [55]. Statistical significance of super (or sub) additive interactions was determined based on whether the 95% confidence interval of the SI crossed 1.0. Given that additive interactions are calculated for risk factors rather than preventative factors [56], additive interactions were only calculated for combinations of variables where both categories were risk factors for unmet needs. Multiplicative interactions were estimated by including interaction terms in the regression model. Interactions were assessed between gender identity and mental/cognitive disabling conditions, occupational gender distribution and mental/cognitive disabling conditions separately. Additive and multiplicative interactions were first assessed in the crude logistic regression model. If they were found to be statistically significant, they were included in the adjusted analysis.

A series of multivariable logistic regression models calculated the odds ratios for women compared to men and individuals with mental/cognitive disabilities compared to individuals without disabilities, as well as individuals in female-dominant occupations compared to gender-neutral and male-dominant occupations, controlling for study covariates. For each of the nested models, all significant variables identified in the crude logistic analysis were included. Included variables were grouped (i.e., individual factors, work context, and job conditions) based on conceptual similarities, each group of variables were manually included in a sequential entry process where groups of variables were added to the model using block-wise forward selection. Data were analyzed using SAS software version 9.4 (SAS Institute Inc., Cary, NC, 2014).

## Results

A total of 2307 employed Canadians completed the survey between May and June 2020. Women, younger respondents, those with lower educational attainment, and those on short-term leave were more likely to be living with mental or cognitive disabilities (see Table 2). Those living with cognitive or mental disabilities reported higher physical work demands, higher mental work demands, lower job control, more job insecurity, more temporary work, more precarious wages, and more vulnerability at work, compared with those without disabilities. Across the three accommodation outcomes, there were significant differences in unmet workplace needs between participants with and without a mental/cognitive condition causing disability. Specifically, 19.0% of participants without a condition causing disability reported unmet workplace flexibility needs compared to 35.9% among participants with a mental/cognitive condition causing disability. Among participants without a condition causing disability, 19.6% reported unmet work modification needs, compared to 34.1% with a mental/cognitive condition causing disability. Of the participants without a condition causing disability 12.2% reported having unmet health benefit needs, compared to 19.9% of participants with a mental/cognitive condition causing disability. At the same time, 53.0% of participants working with a mental/cognitive condition causing disability reported their workplace flexibility accommodation needs met, 40.0% reported work modifications needs met, and 75.6% reported having their health benefits needs met.

**Table 2:** Study Characteristics by Type of Condition causing Disability (n = 2307)

	**No Condition causing Disability**	Mental or Cognitive Condition causing Disability	P-value	
	**(n = 1748)**	**(n = 558)**		
	% (n) / mean (SD)	% (n) / mean (SD)		
INDIVIDUAL FACTORS				
Gender Identity			< 0.001	
Men	54.1% (945)	42.3% (236)		
Women	45.4% (794)	55.0% (307)		
Occupational Gender Distribution			0.16	
Male dominant (> 66% male))	28.6% (499)	32.4% (181)		
Gender neutral (33–66% male)	43.1% (753)	42.3% (236)		
Female dominant (< 33% male)	28.4% (496)	25.3% (141)		
Age (in years)	42.7 (12.2)	39.2 (11.2)	< 0.001	
Education			0.005	
High school diploma or less	6.8% (118)	9.0% (50)		
Some college/university	14.5% (253)	19.0% (106)		
Post-secondary degree/diploma	78.6% (1373)	72.0% (402)		
WORKPLACE CONTEXT				
Union Membership			0.36	
Unionized	35.1% (613)	37.1% (207)		
Non-unionized	64.1% (1121)	61.8% (345)		
Work Schedule			0.096	
Regular daytime schedule	79.8% (1396)	76.2% (425)		
Non-standard work hours	18.9% (331)	22.0% (123)		
Work Hours			0.0002	
Employed full-time	85.3% (1491)	79.9% (446)		
Employed part-time	10.6% (186)	11.8% (66)		
On short-term leave/temporarily laid off	4.0% (70)	8.2% (46)		
Industry			0.55	
Banking, insurance, business, technical, professional	21.7% (380)	22.9% (128)		
Education, health, sciences, art, social sciences, non-profit	32.8% (573)	34.1% (190)		
Government	15.7% (275)	16.7% (93)		
Sales, retail, hospitality	10.7% (187)	10.0% (56)		
Construction, transportation, utilities, manufacturing, agriculture, mining, logging	18.6% (325)	15.6% (87)		
Workplace Size			0.2	
1–99 employees	48.9% (855)	47.9% (267)		
100–500 employees	23.8% (416)	27.4% (153)		
500 + employees	26.5% (463)	24.0% (134)		
JOB CONDITIONS				
Physically Demanding Work (0–4)	1.3 (1.3)	1.6 (1.4)	< 0.001	
Mentally Demanding Work (0–4)	2.9 (1.1)	3.2 (1.0)	< 0.001	
Job Control (0–4)	2.2 (1.1)	2.0 (1.1)	< 0.001	
Job Insecurity (0–4)	1.5 (1.1)	1.8 (1.1)	< 0.001	
Temporariness of Work (0–4)	0.4 (0.6)	0.5 (0.6)	< 0.001	
Precariousness of Wages (0–4)	1.2 (0.8)	1.5 (0.9)	< 0.001	
Vulnerability of Work (0–4)	1.2 (1.0)	1.7 (1.1)	< 0.001	
ACCOMMODATION OUTCOMES				
Workplace Flexibility			< 0.001	
No needs	21.1% (362)	12.9% (70)		
All needs met	59.3% (1018)	53.0% (288)		
Unmet needs	19.6% (337)	34.1% (185)		
Work Modifications			< 0.001	
No needs	39.3% (673)	24.1% (130)		
All needs met	41.7% (714)	40.0% (216)		
Unmet needs	19.0% (326)	35.9% (194)		
Health Benefits			< 0.001	
No needs	10.2% (178)	3.9% (22)		
All needs met	76.5% (1337)	75.6% (422)		
Unmet needs	12.2% (214)	19.9% (111)		

Table 3 presents results for the unadjusted multiplicative (Model 1 and 2) and additive (Model 3 and 4) interaction analysis of (1) gender identity and disability and (2) occupational gender distribution and disability on the three workplace accommodation outcomes (crude and multivariable logistic regression analyses without interactions are presented in Appendices A and B). Results from the multiplicative interaction analysis indicated the interaction between gender identity and disabling conditions and the interaction between occupational gender distribution and disabling conditions were not significant effect modifiers associated with unmet needs for the three workplace accommodations. That is, there was no difference in the effect of gender identity or occupational gender distribution across disability groups on the log scale for any of the outcomes. Results from the crude analysis of the additive interaction indicated that there were no super-additive effects of the interaction between gender identity and disability or occupational gender distribution and disability on any of the workplace accommodation outcomes.

**Table 3:** Crude Multiplicative and Additive Interaction Analysis between Gender Identity and Disabling Conditions and Occupational Gender Distribution and Disabling Conditions on Unmet Needs for Workplace Accommodations

	Unmet needs for Workplace Flexibility	Unmet needs for Work Modifications	Unmet needs for Health Benefits
	(ref = all needs met)	(ref = all needs met)	(ref = all needs met)
**Model 1**: **Multiplicative Interaction Analysis between Gender Identity and Disabling Conditions**	*Type 3 Test P-value*	*Type 3 Test P-value*	*Type 3 Test P-value*
Gender Identity*Disabling Conditions	0.55	0.28	0.54
**Model 2: Multiplicative Interaction Analysis between Occupational Gender Distribution and Disabling Conditions**	*Type 3 Test P-value*	*Type 3 Test P-value*	*Type 3 Test P-value*
Occupational Gender Distribution *Disabling Conditions	0.33	0.54	0.64
**Model3**: **Additive Interaction Analysisbetween Gender Identity and Disabling Conditions**	*Risk Ratio (95% CI)*	*Risk Ratio (95% CI)*	*Risk Ratio (95% CI)*
(ref = men without disabling conditions)			
Gender Identity* Disabling Condition			
Women	**1.53**	1.21	**1.56**
	**(1.20–1.97)**	(0.93–1.58)	**(1.17–2.09)**
Disabling Condition	**1.88**	**1.56**	**1.56**
	**(1.32–2.68)**	**(1.09–2.25)**	**(1.03–2.35)**
Women with mental/cognitive conditions	**2.82**	**2.73**	**2.46**
	**(2.09–3.81)**	**(1.99–3.76)**	**(1.74–3.46)**
Synergy Index	1.29	2.22	1.3
	(0.71–2.35)	(0.92–5.34)	(0.61–2.79)
**Model 4: Additive Interaction Analysis between Occupational Gender Distribution and Disabling Conditions** (ref = individuals in male dominant occupations without disabling conditions)	*Risk Ratio (95% CI)*	*Risk Ratio (95% CI)*	*Risk Ratio (95% CI)*
Occupational Gender Distribution * Disabling Condition			
Female dominant occupations	**1.59**	**1.43**	0.9
	**(1.23–2.07)**	**(1.09–1.88)**	(0.54–1.51)
Disabling Condition	**1.75**	**1.61**	1.04
	**(1.17–2.62)**	**(1.05–2.45)**	(0.77–1.41)
Female do minant occupations with mental/cognitive conditions	**2.92**	**2.48**	**2.11**
	**(2.08–4.12)**	**(1.73–3.56)**	**(1.45–3.06)**
Synergy Index	1.43	1.43	--
	(0.67–3.03)	(0.58–3.56)	

Table 4 presents nested multivariable regression models examining the difference between individuals living with a mental/cognitive condition causing disability and individuals without a condition causing disability in unmet need for workplace flexibility, work modifications, and health benefits. After controlling for individual factors, individuals living with a mental/cognitive disability had higher odds of reporting unmet needs for work modifications, workplace flexibility, and health benefits compared with those with no disability. Including work context variables in addition to individual factors produced similar results. However, controlling for individual factors and job conditions, as well as individual, work context and job conditions yielded no significant differences between people living with or without mental or cognitive conditions causing disabilities in any unmet accommodation needs.

**Table 4:** Odds Ratios for Respondents with Mental or Cognitive Disabilities Versus Respondents without Disabilities across Combinations of nested Logistic Regression models for unmet Accommodation needs

	Unmet needs for Workplace Flexibility (ref = all needs met)	Unmet needs for Work Modifications (ref = all needs met)	Unmet needs for Health Benefits (ref = all needs met)
	*n = 2084*	*n = 2165*	*n = 2182*
**Model 1**: Individuals with mental/cognitive disabilities Vs. Individuals without disabilities	1.94*** (1.55–2.44)	1.93*** (1.52–2.46)	1.57** (1.21–2.05)
**Model 2**: Individuals with mental/cognitive disabilities Vs. Individuals without disabilities + Individual factors	1.1*** (1.36–2.16)	1.83*** (1.44–2.34)	1.37* (1.05–1.80)
**Model 3**: Individuals with mental/cognitive disabilities Vs. Individuals without disabilities + Individual factors + Work Context	1.66*** (1.30–2.11)	1.80*** (1.40–2.31)	1.37* (1.02–1.85)
**Model 4**: Individuals with mental/cognitive disabilities Vs. Individuals without disabilities + Individual factors + Job Conditions	1.05 (0.80–1.38)	1.22 (0.92–1.61)	1.04 (0.76–1.43)
**Model 5**: Individuals with mental/cognitive disabilities Vs. Individuals without disabilities + Individual factors + Work Context + Job Conditions	1.03 (0.78–1.36)	1.25 (0.95–1.65)	1.08 (0.77–1.50)

*p < 0.05, **p < 0.001, ***p < 0.0001

Table 5 presents nested multivariable regression models examining differences by gender identity in unmet needs for workplace flexibility, work modifications, and health benefits. After controlling for individual factors, women had higher odds of reporting unmet needs for work modifications, workplace flexibility, and health benefits compared with men. Controlling for individual factors and work context factors, women were more likely to report unmet needs for work modifications compared to men, but there were no significant differences in unmet needs for workplace flexibility or health benefits by gender identity. After controlling for individual factors and job conditions (Model 4), women were more likely to report having unmet accommodation needs for work modifications and workplace flexibility compared with men. The final model controlled for individual factors, work context, and job conditions. In this model, there were no significant differences between men and women in reporting unmet needs for work modifications and health benefits. After controlling for individual factors, work context, and job conditions, women were more likely to report unmet needs for workplace flexibility compared with men.

**Table 5:** Odds Ratios for Women Versus men Across Combinations of nested Logistic Regression models for unmet Accommodation needs

	Unmet needs for Workplace Flexibility (ref = all needs met)	Unmet needs for Work Modifications (ref = all needs met)	Unmet needs for Health Benefits (ref = all needs met)
	*n = 2084*	*n = 2147*	*n = 2182*
**Model 1**: Women vs. Men	1.66*** (1.34–2.05)	1.39* (1.11–1.73)	1.57** (1.23–2.01)
**Model 2**: Women vs. Men + Individual factors	1.55*** (1.25–1.92)	1.29* (1.03–1.62)	1.53* (1.19–1.96)
**Model 3**: Women vs. Men + Individual factors + Work Context	1.41* (1.11–1.79)	1.18 (0.93–1.51)	1.22 (0.91–1.63)
**Model 4**: Women vs. Men + Individual factors + Job Conditions	1.45* (1.13–1.85)	1.19 (0.92–1.52)	1.43* (1.07–1.91)
**Model 5**: Women vs. Men + Individual factors + Work Context + Job Conditions	1.35* (1.04–1.75)	1.10 (0.84–1.43)	1.27 (0.92–1.76)

*p < 0.05, **p < 0.001, ***p < 0.0001

Table 6 presents nested multivariable regression models examining individuals employed in female-dominant occupations and individuals employed in male-dominant occupations in unmet need for work accommodations. After controlling for individual factors, individuals employed in female-dominant occupations were more likely to report unmet accommodation needs in all three outcomes compared to those employed in male-dominant occupations. After controlling for individual and work context variables, individuals employed in female-dominant occupations were more likely to report unmet accommodation needs for workplace flexibility, work modifications, and health benefits compared with individuals employed in male-dominant occupations. In the final model, after controlling for individual factors, work context, and job conditions, there were no significant differences between those employed in female-dominant occupations and those employed in male-dominant occupations across three types of workplace accommodations.

**Table 6:** Odds Ratios for Respondents Working in Female Dominant Occupations Versus Respondents Working in male Dominant Occupations across Combinations of nested Logistic Regression models for unmet Accommodation needs

	Unmet needs for Workplace Flexibility (ref = all needs met)	Unmet needs for Work Modifications (ref = all needs met)	Unmet needs for Health Benefits (ref = all needs met)
	*n = 2155*	*n = 2147*	*n = 2182*
**Model 1**: Female dominant occupations vs. Male dominant occupations	2.01*** (1.52–2.66)	1.73*** (1.29–2.33)	1.61* (1.15–2.26)
**Model 2**: Female dominant occupations vs. Male dominant occupations + Individual factors	1.62* (1.20–2.19)	1.55* (1.13–2.13)	1.34 (0.93–1.92)
**Model 3**: Female dominant occupations vs. Male dominant occupations + Individual factors + Workplace Context	1.44* (1.02–2.04)	1.42* (1.00–2.02)	1.55* (1.01–2.36)
**Model 4**: Female dominant occupations vs. Male dominant occupations + Individual factors + Job Conditions	1.44 * (1.02–2.02)	1.49* (1.05–2.12)	1.05 (0.69–1.59)
**Model 5**: Female dominant occupations vs. Male dominant occupations + Individual factors + Workplace Context + Job Conditions	1.31 (0.92–1.85)	1.31 (0.89–1.93)	1.21 (0.78–1.87)

*p < 0.05, **p < 0.001, ***p < 0.0001

## Discussion

This study presents novel findings on unmet needs for workplace accommodations and supports in a large sample of Canadian workers living with and without mental/cognitive conditions causing disability that considers a range of job conditions and work context factors. In addition, this research is innovative in being the first to examine the potential interactions between occupational gender distribution and disability on the unmet needs for workplace accommodations and supports. Our findings highlight that, while many people received the workplace accommodations and supports that they reported needing, people living with a mental/cognitive condition and people employed in female-dominant occupations had higher rates of unmet needs compared with those with no disabling conditions and people employed in male-dominant occupations. These differences were largely reduced after considering work context and job conditions. The results are important and point to the need for more focus on workplace environments and ways that some workplaces are able to more successfully accommodate workers with mental health needs, as well as an increased understanding of barriers to support.

Although our study found higher levels of unmet needs among respondents living with mental/cognitive conditions causing disability, many respondents without disabling conditions still had unmet workplace accommodation needs. These results indicate that individuals with and without disabling conditions are not having all their accommodation needs met and that all individuals could benefit from expanded access to accommodation in the workplace. Workplace accommodations are helpful not only for individuals with disabling conditions but have also been shown to be supportive for individuals throughout the life course, for example as they provide care for children or elderly relatives [57, 58]. This is an important finding which stresses the need for employers to be proactive in offering common workplace accommodations, such as work modifications and workplace flexibility, given that individuals with and without mental/cognition conditions may benefit. In light of the high workplace related costs associated with short- or long-term absences from work and return-to-work process, proactively offering accommodations can potentially support employees in reducing use and length absences [18] and better return to work outcomes [59]. Although more research is still needed to quantify the role of accommodations in reducing absenteeism and presenteeism among employees with mental or cognitive disabilities.

Our study found that many workers living with mental/cognitive conditions causing disability, as well as respondents who identify are employed in female-dominant occupations, reported unmet accommodation needs related to flexibility of work and being able to modify the work environment. However, after examining the work context and job conditions, the findings suggested that these differences are likely to be related to the job itself, particularly factors such as precarious work. Jobs associated with contract work, part-time hours, and precarious job conditions such as lower job control, more job insecurity, more precarious wages, and more vulnerability are more likely to be held by people living with mental/cognitive conditions causing disability and women [31–33, 36, 37, 42]. The characteristics of this type of work could also result in barriers to meeting employees’ needs for workplace flexibility and work modifications. For example, the precarious nature of their employment might discourage individuals from requesting accommodations because of a fear of stigma or reprisals [60]. This hesitancy may be intensified among women living with mental health/cognitive conditions causing disability or when working in female-dominated occupations [61, 62]. More research needs to be conducted that investigates the nature of the jobs, especially related to scheduling and flexibility, and whether new types of work, including hybrid working arrangements are introducing greater flexibility into the working lives of people with mental health conditions and women, or whether they are more likely to be employed in jobs that require attendance at a workplace.

Interestingly, women had higher unmet needs for workplace flexibility compared to men, and these differences did not disappear after controlling for work context and job conditions. Future work is needed to replicate these findings. However, this might suggest that there is something unique about the availability of workplace accommodations and supports within jobs held by women that increases the risk of unmet needs, beyond the job conditions and work contexts examined in this study. For example, women are over-represented in jobs that tend to be more client or service focused and therefore can be more difficult to allow for flexibility of location or timing of work. In addition, women are more likely to be employed in positions characterized by precarious work factors which may result in hesitancy in requesting accommodations for fear of reprisals.

Having found that there are unmet needs that might be alleviated with different work contexts, it is important to recognize that there may be structural constraints in the work context that prevent the wide implementation of workplace accommodations, particularly workplace flexibility. This is consistent with prior research conducted on individuals living with physically disabling conditions [16]. While flexibility in work schedule has increased in recent years, mostly due to the COVID-19 pandemic and work from home arrangements [63], it is estimated that only approximately 40% of Canadian jobs can feasibly be done from home [64]. Additionally, jobs that allow the most work from home flexibility tend to be in industries such as finance, insurance, professional, scientific, or technical services and those that allow the least amount of work from home flexibility tend to be in the service industries [64]. Women and people living with mental/cognitive conditions, which are the populations most in need of this accommodation, tend to be underrepresented in the former industries and overrepresented in the latter industries [65, 66]. This may partially explain the findings of this study indicating a higher proportion of unmet needs for women and people with mental/cognitive disabilities. There is a need for future research to explore whether changes to the work environment of different industries and job duties can introduce more workplace flexibility, or other helpful workplace accommodations, to better suit the needs of employees.

This study has several strengths. It used a large sample size of people with and without mental or cognitive disabilities of working age and included results from a comprehensive survey that included multiple factors assessing aspects of work context, job conditions, and individual factors. Second, we used several validated instruments to measure disability which is in alignment with the measures used in large national surveys [49]. Finally, to our knowledge, this is the first paper to examine interactions between occupational gender distribution and disabling conditions on unmet needs for workplace accommodations. It therefore contributes to the expansion of our understanding of the role of gender on work and health.

At the same time, there are study limitations that need to be acknowledged. We tested six interactions, none of which had statistically significant additive interactions. The confidence intervals of the six estimates were, at times, quite wide which implies our study might be underpowered to detect interactions. Future research needs to replicate this research with a larger sample size. Another limitation is the measurement of gender and our being unable to fully capture gender identity. While there were non-binary options in the survey instrument, the number of respondents in these categories was too low to analyze as a separate category. Future research needs to examine the role of gender norms and expression on accommodation and support seeking behaviour in the workplace. More research is also needed to examine the validity and reliability of measures assessing accommodation needs. The items included in the questionnaire were pilot tested and are in alignment with recent research studies assessing workplace accommodations for people experiencing physical and non-physical disabilities [16–19, 29, 67]. However, workplace support policies and practices are evolving, and perceptions of need and availability need further examination.

## Conclusion

In conclusion, our study findings show that, while many people are receiving the workplace accommodations and supports that they need, people living with a mental/cognitive condition and women have higher rates of unmet needs. In addition, many these differences in unmet needs were largely explained by adjustment for work context and job conditions which highlights the need for greater attention to addressing conditions and structural constraints in the workplace environment that prevent the wider adoption of common workplace accommodations and supports, as well as a more detailed understanding of mental health needs, gender, and gendered work.

### Electronic Supplementary Material

Below is the link to the electronic supplementary material.

Supplementary Material 1

## Data Availability

The datasets generated during and/or analysed during the current study are not available upon request.
