# Supplementary material for: Understanding the Unmet Accommodation Needs of People Working with Mental or Cognitive Conditions: The Importance of Gender, Gendered Work, and Employment Factors
Source: J Occup Rehabil. 2023 Oct 25;34(1):251–64. doi: 10.1007/s10926-023-10132-4 (PMC10899322; doi:10.1007/s10926-023-10132-4)
Supplement: Supplementary file 1 — Supplementary Material 1 [file 10926_2023_10132_MOESM1_ESM.docx]

APPENDICES

**Appendix A**: Crude logistic regression for the relationship between study variables and unmet needs for work modifications, workplace flexibility, and health benefits.

|  | **Workplace Flexibility**  **(ref=all needs met)** | **Work Modifications (ref=all needs met)** | **Health Benefits**  **(ref=all needs met)** |
| --- | --- | --- | --- |
| Model 1: main effects | **Unmet needs (n=229)** | **Unmet needs (n=503)** | **Unmet needs (n=131)** |
| INDIVIDUAL FACTORS | | | |
| Gender Identity (ref=men)  Women | 1.66***  (1.34 – 2.05) | 1.39*  (1.11 – 1.73) | 1.56**  (1.22 - 2.00) |
| Age (in years) | 0.98***  (0.97 - 0.99) | 0.98**  (0.97 - 0.99) | 0.99*  (0.98 - 1.00) |
| Education (ref=post-secondary degree or diploma)  High school diploma or less  Some college/university | 1.28  (0.85 - 1.92)  1.53*  (1.15 – 2.02) | 1.02  (0.66 – 1.57)  1.44*  (1.07 - 1.94) | 2.16**  (1.42 - 3.26)  1.64*  (1.19 - 2.25) |
| Type of Condition (ref=no condition causing disability)  Mental/cognitive condition causing disability | 1.94***  (1.55 - 2.44) | 1.93***  (1.52 - 2.46) | 1.57**  (1.21 - 2.05) |
| WORKPLACE CONTEXT | | | |
| Union Membership (ref=unionized)  Non-unionized | 0.61***  (0.49 - 0.76) | 0.87  (0.70 – 1.10) | 2.89***  (2.13 - 3.90) |
| Occupational Gender Distribution (ref= Male dominant (>66% male))  Gender neutral (33-66% male)  Female dominant (< 33% male) | 1.10  (0.85 - 1.44)  2.01***  (1.52 - 2.66) | 1.13  (0.86 - 1.49)  1.73**  (1.29 - 2.33) | 1.46*  (1.06 - 2.00)  1.10  (0.74 - 1.62) |
| Work Schedule (ref=Regular schedule)  Non-standard work schedule | 2.62***  (2.04 – 3.37) | 1.91***  (1.45 – 2.51) | 2.86***  (2.18 - 3.73) |
| Work Hours (ref=employed full-time)  Employed part-time  On short term leave/temporarily laid off | 1.52 *  (1.10 – 2.09)  4.41***  (2.80 – 6.94) | 1.45*  (1.02 – 2.07)  3.53***  (2.13 – 5.86) | 6.62***  (4.78 - 9.16)  4.06***  (2.59 - 6.37) |
| Industry (ref=government)  Banking, insurance, business, technical, professional  Education, health, sciences, art, social sciences, non-profit  Sales, retail, hospitality  Construction, transportation, utilities, manufacturing, agriculture, mining, logging | 0.80  (0.55 - 1.15)  1.74**  (1.26 - 2.41)  2.44***  (1.63 - 3.65)  1.60 *  (1.11 - 2.32) | 1.50 *  (1.03 – 2.19)  2.21***  (1.55 - 3.14)  3.36***  (2.15 – 5.26)  1.83*  (1.22 - 2.76) | 2.18*  (1.28 - 3.72)  3.02***  (1.83 - 4.96)  8.00***  (4.67 - 13.70)  3.21***  (1.89 - 5.46) |
| Workplace Size (ref:500+ employees)  1-99 employees  100-500 employees | 1.30 *  (1.01 - 1.67)  1.16  (0.87 - 1.55) | 1.30  (0.99 - 1.69)  1.20  (0.89 - 1.63) | 3.92***  (2.70 - 5.68)  2.00*  (1.31 - 3.07) |
| JOB CONDITIONS | | | |
| Physically Demanding Work (0-4) | 1.53***  (1.41 – 1.65) | 1.33***  (1.22 – 1.44) | 1.42***  (1.30 – 1.54) |
| Mentally Demanding Work (0-4) | 1.00  (0.90 – 1.10) | 1.06  (0.95 – 1.18) | 0.68***  (0.61 – 0.76) |
| Job Control (0-4) | 0.60***  (0.54 – 0.66) | 0.73***  (0.66 – 0.81) | 0.83**  (0.74 – 0.93) |
| Job Insecurity (0-4) | 1.39***  (1.26 - 1.52) | 1.44***  (1.30 - 1.59) | 1.50***  (1.34 - 1.67) |
| Temporariness of Work (0-4) | 1.17  (0.98 - 1.40) | 1.27*  (1.06 - 1.54) | 2.77***  (2.28 - 3.36) |
| Precariousness of Wages (0-4) | 2.06***  (1.81 - 2.34) | 1.77***  (1.55 - 2.01) | 2.99***  (2.56 - 3.49) |
| Vulnerability of Work (0-4) | 2.90***  (2.56 - 3.27) | 2.95***  (2.59 - 3.36) | 1.48***  (1.31 - 1.66) |

*p<0.05, **p<0.001, ***p<0.0001

**Appendix B:** Multivariable logistic regression for the relationship between individual, work context, and job condition variables and unmet accommodation needs for work modifications, workplace flexibility, and health benefits (n=2307)

|  | **Workplace Flexibility**  **(ref=all needs met)** | **Work Modifications**  **(ref=all needs met)** | **Health Benefits**  **(ref=all needs met)** |
| --- | --- | --- | --- |
|  | *Unmet needs (n=503)* | *Unmet needs (n=229)* | *Unmet needs (n=131)* |
| INDIVIDUAL FACTORS | | | |
| Gender Identity (ref=men)  Women | 1.35 *  (1.04 – 1.75) | 1.10  (0.84 – 1.43) | 1.27  (0.92 – 1.76) |
| Age (in years) | 0.99  (0.98 – 1.00) | 0.99  (0.98 – 1.01) | 1.01  (1.00 – 1.02) |
| Education (ref=post-secondary degree or diploma)  High school diploma or less  Some college/university | --- | --- | --- |
| Type of Condition (ref=no condition causing disability)  Mental or cognitive condition causing disability | 1.03  (0.78 – 1.36) | 1.25  (0.95 – 1.65) | 1.08  (0.77 – 1.50) |
| WORKPLACE CONTEXT | | | |
| Union Membership (ref=unionized)  Non-unionized | 0.65*  (0.49 – 0.85) | --- | 3.50***  (2.39 – 5.13) |
| Occupational Gender Distribution (ref= Male dominant (>66% male))  Gender neutral (33-66% male)  Female dominant (< 33% male) | 0.95  (0.69 – 1.30)  1.31  (0.92 – 1.86) | 1.03  (0.73 – 1.45)  1.31  (0.89 – 1.93) | 1.01  (0.68 – 1.48)  1.21  (0.78 – 1.87) |
| Work Schedule (ref=Regular schedule)  Non-standard work schedule | --- | --- | 0.59*  (0.41 – 0.84) |
| Work Hours (ref=employed full-time)  Employed part-time  On short term leave/temporarily laid off | --- | --- | 2.54***  (1.67 – 3.86)  1.24  (0.71 – 2.16) |
| Industry (ref=government)  Banking, insurance, business, technical, professional  Education, health, sciences, art, social sciences, non-profit  Sales, retail, accommodation  Construction, transportation, utilities, manufacturing, agriculture, mining, logging | --- | 1.58 *  (1.03 – 2.44)  1.66 *  (1.10 – 2.52)  1.77 *  (1.04 – 3.01)  1.38  (0.86 – 2.22) | --- |
| Workplace Size (ref:500+ employees)  1-99 employees  100-500 employees | 1.30  (0.96 – 1.77)  1.10  (0.79 – 1.55) | 1.14  (0.83 – 1.56)  1.12  (0.79 – 1.58) | 2.22*  (1.43 – 3.43)  1.53  (0.94 – 2.50) |
| JOB CONDITIONS | | | |
| Physically Demanding Work (0-4) | 1.32***  (1.20 – 1.46) | 1.17*  (1.05 – 1.30) | 1.26**  (1.12 – 1.42) |
| Mentally Demanding Work (0-4) | 0.96  (0.85 – 1.09) | --- | 0.76***  (0.66 – 0.87) |
| Job Control (0-4) | 0.86 *  (0.76 – 0.97) | --- | --- |
| Job Insecurity (0-4) | 1.13 *  (1.01 – 1.28) | 1.14*  (1.01 – 1.29) | 1.21*  (1.05 – 1.40) |
| Temporariness of Work (0-4) | --- | --- | 2.41***  (1.89 – 3.06) |
| Precariousness of Wages (0-4) | 1.31*  (1.11 – 1.54) | 1.15  (0.97 – 1.36) | 1.71***  (1.40 – 2.09) |
| Vulnerability of Work (0-4) | 2.49***  (2.17 – 2.85) | 2.63***  (2.29 – 3.02) | 1.30**  (1.12 – 1.51) |

*p<0.05, **p<0.001, ***p<0.0001
